# CellFIT: A Cellular Force-Inference Toolkit Using Curvilinear Cell Boundaries

**DOI:** 10.1371/journal.pone.0099116

**Published:** 2014-06-12

**Authors:** G. Wayne Brodland, Jim H. Veldhuis, Steven Kim, Matthew Perrone, David Mashburn, M. Shane Hutson

**Affiliations:** 1 Department of Civil and Environmental Engineering, University of Waterloo, Waterloo, Ontario, Canada; 2 Department of Physics and Astronomy, Vanderbilt University, Nashville, Tennessee, United States of America; 3 Department of Biological Sciences, Vanderbilt University, Nashville, Tennessee, United States of America; 4 Vanderbilt Institute for Integrative Biosystem Research & Education, Vanderbilt University, Nashville, Tennessee, United States of America; University of Cambridge, United Kingdom

## Abstract

Mechanical forces play a key role in a wide range of biological processes, from embryogenesis to cancer metastasis, and there is considerable interest in the intuitive question, “Can cellular forces be inferred from cell shapes?” Although several groups have posited affirmative answers to this stimulating question, nagging issues remained regarding equation structure, solution uniqueness and noise sensitivity. Here we show that the mechanical and mathematical factors behind these issues can be resolved by using curved cell edges rather than straight ones. We present a new package of force-inference equations and assessment tools and denote this new package CellFIT, the Cellular Force Inference Toolkit. In this approach, cells in an image are segmented and equilibrium equations are constructed for each triple junction based solely on edge tensions and the limiting angles at which edges approach each junction. The resulting system of tension equations is generally overdetermined. As a result, solutions can be obtained even when a modest number of edges need to be removed from the analysis due to short length, poor definition, image clarity or other factors. Solving these equations yields a set of relative edge tensions whose scaling must be determined from data external to the image. In cases where intracellular pressures are also of interest, Laplace equations are constructed to relate the edge tensions, curvatures and cellular pressure differences. That system is also generally overdetermined and its solution yields a set of pressures whose offset requires reference to the surrounding medium, an open wound, or information external to the image. We show that condition numbers, residual analyses and standard errors can provide confidence information about the inferred forces and pressures. Application of CellFIT to several live and fixed biological tissues reveals considerable force variability within a cell population, significant differences between populations and elevated tensions along heterotypic boundaries.

## Introduction

The shapes and movements of cells and tissues are crucial to a wide range of biological processes – including embryogenesis, wound healing, cancer metastasis and tissue engineering [1]–[15] – but relatively little is known about the underlying mechanical forces. Clearly, knowledge of the forces driving these processes is a fundamental part of a complete understanding. Without it, we will not only struggle to correctly figure out the basic mechanics of cell and tissue reshaping, but we will have little hope of properly identifying the effects that forces have on mitosis, gene expression and differentiation [16].

A large number of experimental techniques have been developed to obtain information about the forces at work in cells. Some are applicable only to cells that reside on the surface of a mass or in a monolayer epithelium directly accessible to physical contact. These techniques, some more historical than others, include thin glass rods inserted to apply forces or constrain natural movements [17], atomic force microscopes that exert known forces and measure associated displacements [18], micropipette aspirations that yield surface tensions [19], and substrate deformation techniques that measure traction forces [15], [20]. Some of these techniques can be used to obtain force or traction maps, but these powerful approaches are not applicable to the interiors of cell masses or to *in vivo* tissues protected by a requisite layer such as a vitelline membrane. Thus, several other techniques have been developed for *in vivo* measurements, including magnetic cytometry in which magnetic forces are applied to inserted ferrous particles [21], morphological techniques based on the shapes of inserted oil droplets [16], optical tweezers that exert forces on endogenous or injected particles with different refractive indices [22], FRET techniques that aim to report deformations and forces [23], [24] by optical means and laser ablation techniques based on recoil rates [12]. All of these experimental techniques provide force information limited to specific locations and times. One could theoretically construct detailed spatial and temporal force maps by collating data from multiple specimens, but animal-to-animal variations make such approaches impractical.

Computational models have also provided a great deal of information about cell- and tissue-level forces [25]–[30]. These models have typically been used in a forward manner, where a user specifies the forces at work in a particular aggregate of cells or other system under study and uses the model to predict the resulting cell shapes and motions. The time-consuming challenge of this approach is figuring out the forces needed to produce a particular morphological outcome. Even when an appropriate set of forces is found, uniqueness is not guaranteed; other sets of driving forces might be able to produce the same outcome. We were able to show that under suitable circumstances and with appropriate side conditions, model equations that calculate motions from forces can be inverted and used to directly estimate forces from shapes and motions [31], [32]. This understanding was the basis of Video Force Microscopy (VFM), a technique that allowed us to construct detailed maps of the dynamic sub-cellular forces driving ventral furrow formation in *Drosophila.*

VFM showed that the initial stages of ventral furrow formation were driven by apical constrictions that were focused on the invagination site, that these tensions arose smoothly over time and that they varied smoothly with medio-lateral position. These findings were unlike the step functions often assumed explicitly or tacitly in conceptual and computational models [33]. VFM also rather unexpectedly showed the presence of moderate and relatively uniform contractions along the basal surface of the dorsal and lateral ectoderm – sites well removed from the location of furrow formation. It further revealed significant tensions along the cell membranes that run across the thickness of the epithelium near the ventral midline, especially during the latter stages of furrow formation. When applied to mutant embryos, VFM showed that the driving forces were affected in specific ways consistent with the observed motion and shape irregularities. Subsequent computational modeling confirmed the validity and necessity of the various force systems revealed by VFM [34].

Unfortunately, attempts to apply this promising technique to wound healing and other in-plane motions led to unexpected challenges such as high sensitivity to noise, including that produced by image digitization. A number of groups, including ours, made headway on the in-plane force-inference problem by using more advanced solvers and modified assumptions about the forces present. For example, Chiou et al. reduced the number of unknown parameters by assuming either that all cells had the same intracellular pressure or that each cell had a single cortical tension that contributed equally to the edge tension along each of that cell's interfaces [35]. The subsequent comprehensive study of Ishihara and Sugimura showed that a Bayesian solver could address general stability issues and the shortage of equations that arises for planar assemblies of cells represented by minimal polygons [36]. Shortly thereafter, a detailed comparative paper evaluated a number of different approaches and combinations of assumptions and provided a summary of the state of the art [37].

The key insight that led to the work reported here, was the realization that substantially-improved equations could be formulated if cell boundaries were treated as being curved, even if only slightly. Nearly all prior force-inference studies, with the exception of some on bubble rafts [38], used a straight edge approximation. This seemingly subtle difference completely changes the nature of the governing equations. It overcomes the equation shortage often encountered in the past, brings redundancy and stability to the assembled matrix equations and reduces noise sensitivity by at least an order of magnitude. Here we describe this new approach, its basic equations and the form of its associated matrices. We show that the tension equations are solvable independent of the pressure equations, and we present mathematical tools for assessing the quality of the resulting solutions.

**Figure 1:**
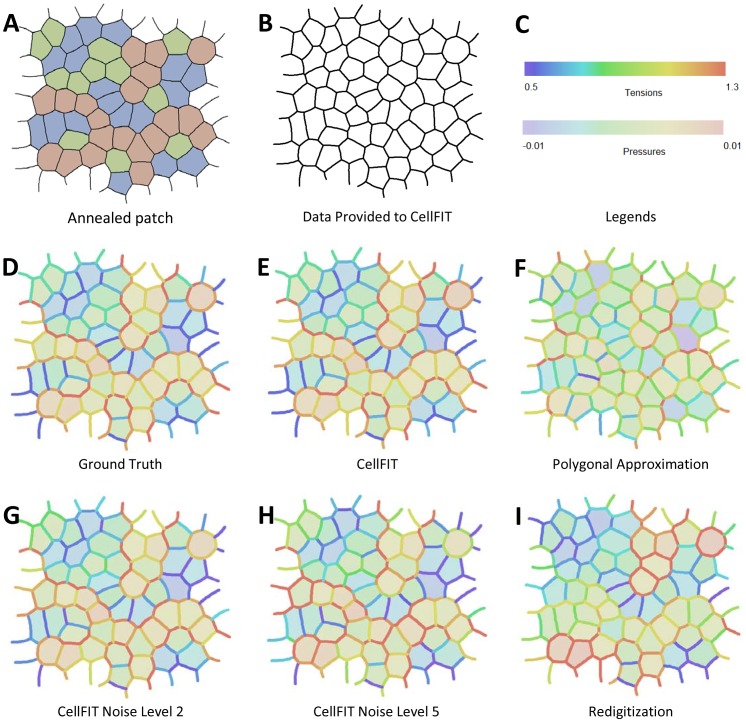
A synthetic planar aggregate and its analysis by CellFIT. (**A**) shows a representative region consisting of 50 complete cells, 177 complete edges, and 30 partial cells and corresponding incomplete edges taken from a larger aggregate. Its cells were assigned to one of three types, as indicated by coloured shading and a finite element model was used to determine its annealed state, as shown. The edge tensions were assigned the following values according to their type 5∶6∶7∶10∶11∶12. (**B**) shows the topological and geometric information provided to the CellFIT algorithms. The spectra shown in (**C**) provide legends for the tension and pressure colours used in the remaining parts of the figure. (**D**) shows the ground truth Standard Tensions and Pressures as determined by the FE annealing process, and extracted using Equation 16 and its associated text. (**E**) shows the values that the standard Polyarc version of CellFIT calculates based solely on the data in (B). (**F**) shows the results obtained by calculating the angles at triple junctions using straight edges only. (**G**) and (**H**) show CellFIT results when noise levels of 2 or 5 are respectively introduced into the CellFIT input data. A noise level of *x* corresponds to *x* degrees of angle error and *x*% curvature error. (**I**) shows CellFIT results obtained after the original mesh was converted into an image and redigitized before analysis.

In this study, we focus on patches of cells extracted from larger cell sheets (Fig. 1A). Consistent with a number of other groups [35]–[37], we assume an underlying model of cell mechanics in which cell shape is governed solely by interfacial tensions that are uniform between each pair of adjacent triple junctions and intracellular pressures that are uniform within each cell. We further assume that motions are sufficiently slow as to make viscous forces negligible, a quasi-static approximation. However, in contrast with previous approaches, we do not approximate cell edges as straight, but instead allow curvature – connecting each pair of adjacent triple junctions with an arc. A cell is thus not represented by a polygon, but by a polyarc. To distinguish this new approach from VFM, we refer to it and the associated tools for assessing solution quality as the Cellular Force-Inference Toolkit or CellFIT. Within this paper, CellFIT by itself implies the use of a polyarc cell representation and variants, such as polygon CellFIT, are so indicated. The term triple junction (TJ) is used widely herein for simplicity and because it is by far the most common kind of junction, however, many of the comments made regarding it have obvious parallels that apply equally well to junctions of different order.

As in VFM, CellFIT begins by determining cell shapes in an image using a combination of automated segmentation and manual tracing. A circular arc is then fit to the pixels corresponding to each cell-cell interface to estimate the edge curvature and the angles at which the edge approaches its triple junctions. This information is used to construct the governing equations. Angles determined by this method may appear only modestly different from those associated with the straight edges of minimal polygons, but the differences affect the assembled equations enough to significantly alter the calculated tensions. Equation sets for both the edge tensions and for the gauge pressures are generally overdetermined, even for meshes that are “open” or have “ragged” edges. This allows a limited number of TJs to be excluded from analysis if one or more of their edge approach angles is in question, e.g., if an edge is extremely short or is not well defined in the image. The edge tension equations are solved first and the solution scaled to actual tensions using data external to the analyzed image. Then, if intracellular pressures are also of interest, Laplace equations are written for each cell edge so as to relate the previously determined edge tensions to the edge curvatures and pressure differences. These equations are assembled and solved to within a pressure offset. Like the tension scaling above, this pressure offset must be obtained from external information. We find that solutions based on the curved edges of polyarc cells are considerably less sensitive to noise than are those based on polygonal cells.

We show that all possible combinations of pressures and tensions consistent with a given image can be built by suitably combining and scaling a single set of normalized tensions and its associated zero-mean gauge pressures – so-called Standard Tensions and Standard Pressures. We also present a number of tools for assessing solution quality: matrix condition numbers, residual plots, and uncertainty estimates based on standard errors.

When applied to images of epithelia, CellFIT yields results consistent with available experimental measurements. However, it goes beyond these measurements to provide insights into force variability within single tissues, force differences between adjacent tissues and elevated tensions along boundaries between tissues.

## Equation Formulation

Consider a contiguous planar monolayer (Fig. 1A), whose three-dimensional form could be produced by extruding its planform normal to the plane of the sheet. The edges of its cells are allowed to be curvilinear in the plane of the sheet, and tensions along those edges are assumed to be uniform within each edge, but to vary from one edge to another. As in other studies, these cell edge tensions or “interfacial tensions” are assumed to arise from the combined action of actomyosin contractions, membrane tensions and cell-cell adhesion systems [27], [39]. While the contractions and tensions tend to shorten the cell edge, the adhesion forces tend to lengthen it. Here, we report the net contractile force, and call it the effective edge tension or simply edge tension *γ*. The mechanical effect of forces transverse to the sheet could also be analyzed [40], but they are not considered here.

In addition, we assume that isotropic tensions act along the apical and basal surfaces of the cells, generating further forces in the plane of the sheet [40], [41]. Within any given cell, these contractions would be mechanically analogous, but opposite in direction to any intracellular pressures that might be present. Here we consider the net isotropic expansion force as a positive effective intracellular pressure or simply intracellular pressure. Cell deformations are assumed to occur sufficiently slowly that viscous forces are negligible and the cell can be considered quasi-static [37], a simplification compared to VFM. As a result of this simplification, the present formulation is not appropriate for analyzing laser ablation recoil or other rapid motions where viscous forces may be significant. Cells may also carry tractions, crawling or friction forces between themselves and a subjacent or overlying structure [14], [42], but these mechanical effects are beyond the scope of the present study.

Instead, the present analysis deals only with in-plane forces, motions and shapes, and it considers them to be governed exclusively by equivalent edge tensions and intracellular pressures. This approach is consistent with other recent studies of cell sheets [35]–[37], and it does not hamper future enhancement or generalization of the equations presented.

**Figure 2:**
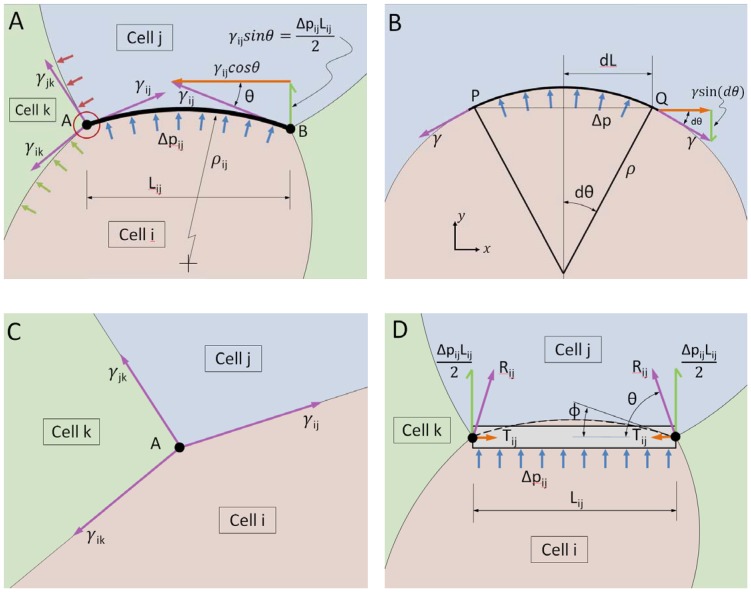
Equilibrium considerations. (**A**) shows a curved cell edge and the forces acting on it, while (**B**) shows how edge tension, curvature and pressure are related. Specifically, the pressure difference Δ*p* generates a force 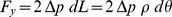 in the *y*-direction. This force must be just balanced by the vertical components of the tension γ. Thus we have that 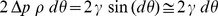 which, when simplified, gives 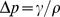, the Laplace equation. (**C**) shows the forces that act at a typical triple junction, while (**D**) shows the forces that act on an edge that is constrained to remain straight by beam action, as described in the text.



Next, consider the curved cell boundary shown schematically in Fig. 2A. Cell-cell interfaces like this behave mechanically like a membrane, in the engineering sense of the word. In contrast to many other kinds of structural elements, membranes carry no bending or shear stress [43] and they rely on tensions along their shape to carry load, typically changing geometry in order to carry newly-applied loads. The interface shown in Fig. 2A between cells *i* and *j* is assumed to carry a tension *γ_ij_*, and to sustain a pressure difference

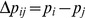
(1)that arises from the difference between the intracellular pressures *p_i_* and *p_j_* in cells *i* and *j*, respectively. The relationship that must exist between pressure *Δp_ij_*, radius of curvature *ρ_ij_* and tension for a portion of a membrane to remain in equilibrium is easy to derive, as shown in Fig 2B and its caption, and the resulting so-called Laplace equation, is 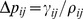
(2)where the radius of curvature *ρ_ij_*, the multiplicative inverse of the membrane curvature, is considered positive when the *i-j* edge is convex into cell *j*.

The cell membrane behaves rather like a sail, which is necessarily bowed when pressure differences arise between its sides as a consequence of wind action. The greater the pressure difference, the greater the tension in the fabric of the sail for a given geometry. Furthermore, as the rigging is tightened, the force in the sail increases and its curvature reduces. In the special case where there is no pressure difference across the sail, the sail would have negligible curvature but still carry tension because the rigging is tight. As in a sail, we assume that the tension along any particular cell edge is sensibly constant. If loads with force components tangent to the sail or membrane were applied, this assumption would need to be modified.

Another set of equations can be constructed by noting that the vector sum of the forces applied to each TJ must add to zero for it to be in equilibrium (Fig. 2C). Just as a sail attached to a mast pulls in the direction of the final limiting angle at which the sail cloth approaches the mast, the vector force **γ** *_ij_* of a cell edge tension acting on a TJ must be away from the TJ and tangent to the edge as it approaches the TJ. For any particular TJ to be in equilibrium, the adjacent cell edges must satisfy the force balance equation 
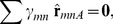
(3)where the unit vectors 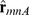 are constructed tangent to the limiting angle at which the membrane along the boundary between cells *m* and *n* approaches the *A_th_* triple junction and pointing away from the junction, and the summation is carried out over all edges that connect to that TJ. The *γ_mn_* values are the corresponding unknown membrane tensions. For illustrative purposes, Fig. 2C shows three such cell edges, but there could be more, as at “quad” junctions and rosettes [44].

Equation 3 may seem oversimplified, not taking pressure forces into proper account. In fact it is complete, as can be argued by considering Fig 2C to represent a very small region around the TJ labeled A, as suggested by the small red circle in Fig. 2A. As the region of interest is made smaller, the length over which pressure forces normal to any given membrane or sail cloth can act becomes vanishingly small and since its contribution is equal to pressure times length, that contribution becomes negligible. Another way to resolve the seeming paradox is to consider a free body diagram of increasing size. As the area considered in the diagram is made larger, the direction of the membrane (or sail) at the edge of the diagram changes. One can show using a figure similar to Fig. 2B, that as the boundary of the free body diagram is expanded, the direction of the tension vector **γ** at the cut edge of the sail changes in such a way that its contribution to the net force at the TJ exactly counterbalances the growing effect of the pressure force as it acts on an increasing area.

If, rather than being curved, cell edges are assumed to be straight (Fig. 2D), then they must act as beams (rather like the mast of a sail boat), carrying both bending and shear in order to remain straight [45]. In that case, pressure loading *Δp_ij_* gives rise to shear forces of magnitude *Δp_ij_ L_ij_/2* at each end of the beam, as shown. The beam can also carry tension *T_ij_* as shown. In VFM, as in other studies that assume straight edges [31], [35]–[37], the pressure *Δp_ij_* and tension *T_ij_* are treated as independent variables and their vector sum **R** *_ij_* may not be tangent to the membrane at the TJ, although it should be. One situation where this non-alignment is not a problem, however, is when the edges of cells are essentially perpendicular to each other as was the case when VFM was used to study ventral furrow formation [31]. The inconsistency noted above is the apparent reason that straight edge-based approaches tend to encounter computational challenges such as high noise sensitivity. They also lack pressure equations (Equation 2) and can thus have more unknowns than equations.

**Figure 3:**
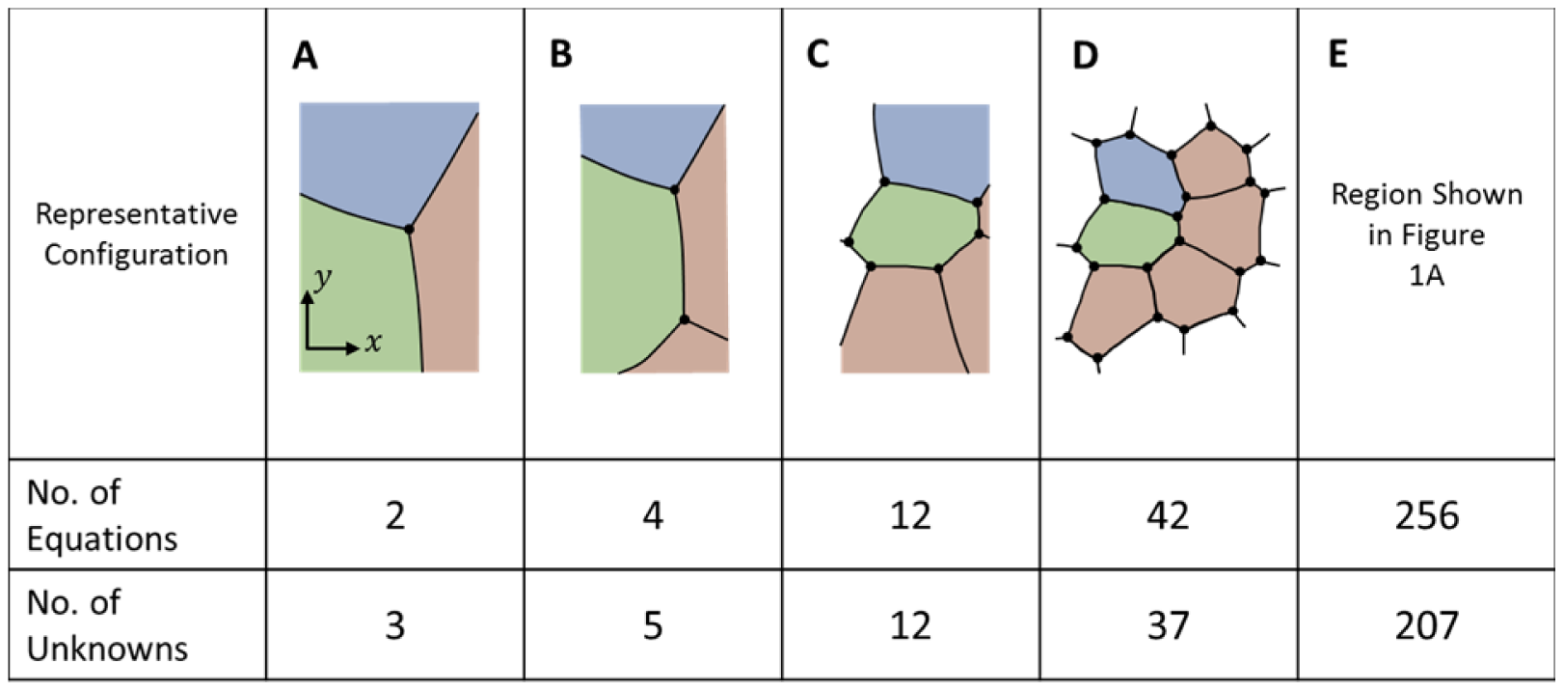
The tension equations are generally overdetermined. Geometric considerations show that the tension equations are, within a scale factor (see text), adequately determined (**A**, and **B**). When a full cell is enclosed (**C**) and as more cells become fully enclosed, they generally become increasingly overdetermined (**D** and **E**), though scaling information external to the image is still needed.

Equation 3 can be written for any single TJ (Fig. 3A), and each such analysis provides two equations (one in each of the *x*- and *y*-directions) with three unknowns (the three interfacial tensions, *γ_mn_*). Although there is not enough information to solve the equations in the conventional sense, each equation set allows the ratios of the three tensions that act on it to be determined, but does not allow their magnitudes to be found. If two triple junctions are adjacent to each other (Fig. 3B), one could set up equations for each junction and, as is done here, assume that the tension in each boundary remains constant along its length. Introducing this assumption means that each additional simply-connected TJ adds two more equations and only two more unknown tensions. When the last TJ needed to complete the perimeter of any particular cell is added (Fig. 3C), two more equations can be written, but only one more new edge is added. As more cells are completed, the equation set becomes increasingly overdetermined. Nonetheless, external information is still needed to scale its solution since the equation set is homogeneous. The overdetermined nature of the resulting equations (Figs 3D and 3E) tends to reduce its sensitivity to any errors in the equilibrium equations from which it is built.

Sometimes, one or more of the cell edges in an image is short, poorly imaged, crenulated or incomplete, making it difficult or impossible to confidently ascertain the angle at which those edges approach one or both of the junctions to which they are attached. The system of tension equations is overdetermined sufficiently to allow exclusion of a limited number of these problematic edges. Doing so requires excluding the *x*- and *y*-force balances associated with the endpoint junctions of the excluded edges, as if those junctions did not exist. The number of useable equilibrium equations then becomes
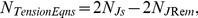
(4)where *N_Js_* is the number of triple, quad or other types of junctions present before any edges are removed and *N_JRem_* is the number junctions removed because they were associated with one or more removed edges. For consistency, all of the parameters appearing on the right hand side of tally Equations 4, 5, 9 and 10 are based on the pre-removal geometry. The number of boundaries whose tensions must be found is 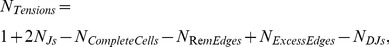
(5)where *N_CompleteCells_* is the number of fully-surrounded cells and *N_ExcessEdges_* is the total number of attachments in excess of 3 per junction over the grid. If there were 4 quad junctions and one rosette with 6 edges, *N_ExcessEdges_* would equal 1+1+1+1+3 = 7. The number of edges removed in *N_RemEdges_*. As suggested by Fig. 3, all triple junctions, including those along the perimeter are included in the count for *N_Js_*, and all of the stub (truncated) edges are included in *N_Tensions_*. Cells in contact with the medium may have only two edges at a given junction and the number of those junctions is denoted *N_DJs_*. Some of the relationships outlined here may appear inconsistent with Euler's formula but that is a consequence of the presented equations allowing for stub edges. Comparison of Equations 4 and 5 shows that as long as the numbers of nodes affected by deleted edges *N_JRem_* and the number of extra attachments *N_ExcessEdges_* are not too large, there will be more equations than unknowns. The tensions in the stub edges are calculated because no particular advantage is realized by removing them and their associated junction equilibrium equations from the analysis. One of the purposes of Equations 4 and 5 and the other tally equations reported here is to provide insight into how the number of equations and unknowns is affected by geometric details of the area studied, information that could be useful to manual or automated schemes for choosing such regions strategically.

To facilitate their solution, the Tension Equations are written in matrix form,
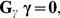
(6)where each of the paired rows in the *N_TensionEqns_* by *N_Tensions_* matrix **G** *_γ_* contains 3 (or more for quad or rosette junctions, or fewer for double junctions) cosines or sines, according to whether that row relates to the equilibrium equation for the *x*- or *y*-direction, respectively. The vector **γ** is a list of length *N_Tensions_* containing the surface tension magnitudes *γ_ij_*. Equation 6 is homogeneous and so as to avoid its natural solution **γ**  =  **0** and obtain a meaningful ratio between the tensions, we construct and solve the constrained least-squares equation system
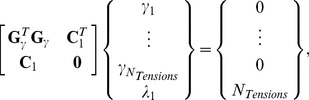
(7)where
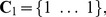
(8)which imposes the condition 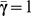, where the overbar indicates taking the mean of the elements in the vector, and *λ_1_* is the Lagrange multiplier associated with this constraint. If a particular tension in a specific edge is desired, less bias is introduced by scaling the above solution than by specifying that tension as a constraint using a modified **C**_1_. Singular value decomposition, least absolute error and logarithmic solvers [46], [47] can also be used to solve Equation 6, and all three approaches appear to generally give similar results. Bayesian solvers [36] and other kinds of sophisticated approaches may be more complex than necessary for solving these simple, well-conditioned equations. The result of this solution process is a set of tensions for all of the *N_Tensions_* edges, that is, for all complete or partial edges in the region of interest less any purposely removed edges. In order to scale the tensions so that their magnitudes are correct in a particular application, external information, such as the tension acting along a particular edge, is needed.

**Figure 4:**
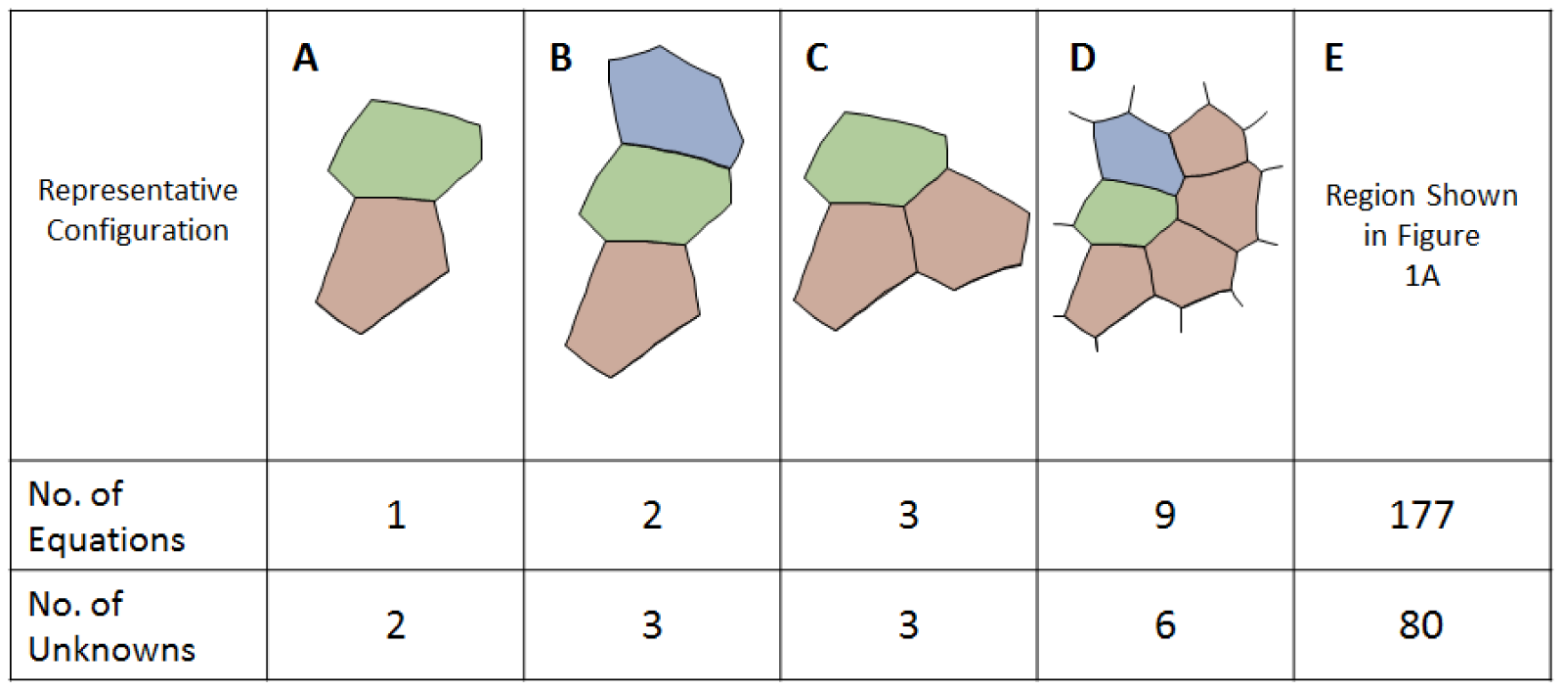
The pressure equations are generally overdetermined. As the number of cells increases and cells acquire multiple neighbours, the Pressure Equations become increasingly well determined (**A** to **E**). Even when the system is seemingly overdetermined (**D** and **E**), external data still is needed to ascertain the pressure offset (see text).



Once the vector of edge tensions **γ** is known in scaled or unscaled form, the effective intracellular pressures can be calculated. For these calculations, the number of available equations is taken to be
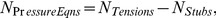
(9)where *N_Stubs_* is the number of stub edges along the perimeter of the region of interest. The stub edges are not used for pressure calculations in the present analysis because there are otherwise ample equations available and because stub edges may not be sufficiently long to provide reliable curvature information. The number of unknown pressures is equal to
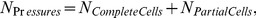
(10)where *N_PartialCells_* is the number of partial cells along the perimeter of the patch. If the region of interest is in contact with medium at one or more locations, that medium is considered a single partial perimeter cell in calculating *N_PartialCells_*. In general, the Pressure Equations are overdetermined (Fig. 4) and well-conditioned, and they can be written in the form

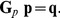
(11)Each row of Equation 11 represents one pressure difference equation. The left side of Equation 11 is a calculation of *Δp_ij_* using Equation 1, and each row of the *N_PressureEqns_* by *N_Pressures_* matrix **G** *_p_* contains two non-zero entries, a 1 and a -1 according to which pressures are involved in Equation 1 and their respective signs. The **q** on the right side of Equation 11 calculates *Δp_ij_* using Equation 2, and its entries are a listing of the ratios 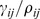 in the order that corresponds to the matrix equations on the left side.

Each of the equations involves a pressure difference and further information, such as an assumption that the medium or wound pressure is considered zero, is needed to establish a definitive pressure offset. A constrained least-squares system 
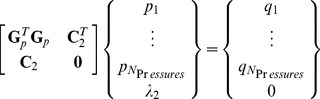
(12)where
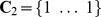
(13)and *λ_2_* is the Lagrange multiplier associated with the constraint 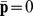. Although Equations 6 and 11 appear similar in form, they are actually quite different in character. In general, only the former is homogeneous and only the latter is rank deficient. As a result, one might use different kinds of solvers for each. When Equations 6 and 11 were combined into a single coupled least squares system and solved together, as part of our study, no particular advantages were observed.

It is worthwhile to consider the meaning of these equations and their solutions. In actuality, the tension balances (Equation 3) and Laplace pressure equations (Equation 2) imply an underlying conceptual model in which cell shapes are determined exclusively by edge tensions and intracellular pressures. Under that assumption, a well-defined mapping exists from cell-level forces to cell shape. The converse relationship is almost as well determined, but the scaling factor for the tensions and the pressure offset cannot be determined from an image alone. Indeed, an entire family of related force sets would produce the same final geometry. Nonetheless, CellFIT returns valuable spatial and temporal maps of the relative forces. This situation is somewhat different from that of VFM, which assumes the presence of non-negligible viscous forces of known form that provide a non-zero right hand side for its equivalent of Equation 6 [31]. As a result, the VFM equation system was non-homogeneous; given an estimate of cell viscosity, VFM could solve for a unique set of tensions and pressures. Incorporating viscous forces will thus be an important future extension of CellFIT.

To better highlight the shape-force relationship that exists when viscous forces are neglected, we propose a canonical form for the CellFIT solutions, a so-called General Solution that encompasses all solutions consistent with a particular image. The General Solution is constructed from a set of Standard Tensions and Standard Pressures. We define the Standard Tensions, denoted **γ** ^*^, as the solution to Equation 6 that is scaled to a mean of one. Alternative scaling based on the L_2_ norm is possible, but mean scaling is a natural consequence of finding Standard Tensions using Equations 7 and 8. The Standard Pressures **p** ^*^ are found by solving Equation 11 with the tensions set to **γ** ^*^.

All solutions consistent with a given image are then given by
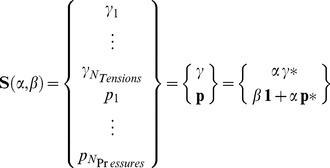
(16)where 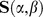 is the so-called General Solution for that image. A unique solution, corresponding to particular values of the unknown scaling factors *α* and *β*, can be found using two external pieces of information such as one edge tension and one reference pressure or two suitable pressures. Physically, the parameter *α* corresponds to the average edge tension while *β* corresponds to the average intracellular pressure. In constructing these solutions, it should be noted that the relationship between the Standard Tensions and Standard Pressures will depend on the image scale since their units are different. Furthermore, the General Solution, Standard Tensions and Standard Pressures can be constructed easily from any mutually consistent pair of tensions and pressures **γ** and **p**, respectively, by carrying out operations inverse to those described by Equation 16.

If groups of cells are connected to each other by at least one edge, or a chain of edges, then a single Tension Equation (Equation 6 or 7) can be constructed for all of them and the relative edge tensions are knowable across the fully connected mesh. Otherwise, separate Tension Equations must be solved and the mechanics of the two regions cannot be linked using information from the image. Separated regions could arise, for example, if a band of the image was out of focus or otherwise marred or if a strip of cells moved out of the imaging plane due to a process such as furrow formation. Likewise, the criteria for a single Pressure Equation (Equation 11 or 12) to apply to groups of cells are (1) that a single Tension Equation is applicable and (2) that the groups are connected by at least one chain of cells with known tensions and curvatures between neighbouring cells along the entire chain. The quality of the relative tension and pressure estimates between two regions will be better if multiple connecting chains exist and if the chains are short, interconnected and multiple cells wide.

If tractions or additional kinds of forces were assumed to act, more complex equations would be required, but such situations are beyond the scope of the present study.

## Validation and Characterization Using Synthetic Data

In order to test and validate the algorithms, they were applied to hundreds of synthetic cell patches for which ground truth values were known. These model epithelia were generated by creating Voronoi tesselations, converting them into finite element (FE) meshes with assigned edge loads and running the FE code until further movement ceased [48], [49]. Unlike many previous FE models, this one had intermediate nodes along each cell edge so that the edges could take their natural curved shapes, and an average of approximately 4 intermediate nodes per edge was found to be sufficient. The annealing process ensured that the resulting meshes were in static equilibrium, a condition that would not be true of raw Voronoi tessellations or arbitrary meshes. In order to generate a model patch with terraced edge forces so that the calculated edge tensions could be more readily evaluated visually, the cells in a large patch were assigned randomly to one of three cell types (Fig. 1A), giving rise to 6 interface types with their corresponding discrete edge tensions. The region shown was extracted from a large patch designed to minimize any boundary effects on the region of interest and so as to produce “frayed” edges rather than the easier to analyze smooth ones that characterize isolated clumps of cells that are surrounded by medium. All force information was stripped from the FE output and only the mesh geometry (Fig. 1B) was passed to the CellFIT algorithm.

CellFIT extracts three kinds of information from the image: cell topology, Young angles and edge curvatures. An algorithm was written to fit a circular arc to all of the nodes along each edge, and when this approach was used, the Young angles were based on the angle of each arc at the point where it made its closest approach to the triple junction. Figs 1D-I show various solutions, and the legend spectra in C apply to all of them. The normalized ground truth tensions and pressures (overlaid on each other in Fig. 1D) resulting from the FE annealing process are essentially indistinguishable from the Standard Solutions found by CellFIT (Fig. 1E). As shown in Table 1, the RMS tension and pressure differences are only 0.7% of the mean tension and 12.5% of the RMS pressure (note that RMS pressure is used because the mean pressure is arbitrary and set to zero in the Standard Solution). Eliminating the three short edges that contained no intermediate nodes reduced both errors to less than 0.3%. In contrast, if the Young angles were calculated using a multi-segmented line approximation to each edge and the angles of the edge segments closest to the TJs were used, the tension and pressure errors increased to 7.4% and 13.4%, respectively. Note that the relative size of the tension and pressure errors shown here and elsewhere in this paper does not necessarily indicate a greater error in the pressures, since the two values are normalized differently. If the angles were instead based on a minimal polygon representation (Fig. 1F) – i.e., one in which the edge from each TJ to the next is treated as a single straight line – the tension and pressure errors increase, respectively, to 27.1% and 25.0%. In all cases, CellFIT performs best when the limiting angles are determined most accurately.

**Table 1:** Normalized Tension and Pressure Errors. The RMS errors in the inferred tensions 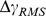 and pressures 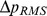 have been normalized to the mean ground truth tension 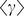 and RMS ground truth pressure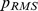, respectively, and reported as a percentage.

	No Noise		Noise 1		Noise 2		Noise 5	
Method	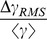	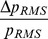	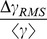	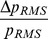	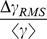	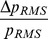	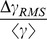	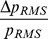
CellFIT – polyarcs	0.7	12.5	3.5±0.7	13.1±0.9	6.8±1.3	14.6±2.1	17.3±3.5	23.5±5.2
CellFIT – polyarcs, short edges removed	0.1	0.3	3.7±0.7	4.3±0.9	7.3±1.3	8.8±2.1	19.6±4.2	24.7±8.2
CellFIT – angles from closest segment	7.4	13.4	8.1±0.5	13.7±0.9	9.9±1.0	14.9±1.8	18.1±2.8	22.9±4.6
CellFIT – angles from minimal polygons	27.1	25.0	27.3±0.5	25.0±0.9	27.9±1.0	25.9±1.8	31.7±2.5	31.1±4.6
Bayesian – minimal polygons	23.8	92.9	24.1±0.4	94.0±1.5	25.1±0.4	98.0±1.9	26.5±0.4	102±3.0

For CellFIT noise analyses, Gaussian-distributed errors of the specified RMS magnitude were introduced into the limiting edge angles and edge curvatures before application of Equations 7 and 12. Noise of level *x* corresponds to introduced RMS angular errors of x degrees and curvature errors of *x* percent of the true values. For noise analysis of Bayesian force inference, which treats cells as minimal polygons, Gaussian-distributed errors were added to the triple-junction coordinates. The RMS magnitude of these coordinate errors was chosen to yield RMS angular errors of *x* degrees. Because introduced tension and pressure errors can produce different outcomes from one run to the next, 100 runs were carried out for each noise level using different random seeds. The statistical properties of those ensembles are reported.

Using this same data, we also examined the performance of VFM [31] and a Bayesian force-inference method [36], [37], both using minimal polygon representations. With such a minimal representation, the VFM system of equations was under-determined and could not be solved (using ordinary least squares regression). Bayesian force inference has a similarly under-determined system of equations, but can find a solution using two priors: Gaussian-distributed edge tensions with a mean of one and pressures with a mean of zero [37]. The Bayesian inference method did not perform as well as CellFIT, yielding errors of 23.8% for the tensions and 92.9% for the pressures (Table 1). The larger tension error is similar to that found when CellFIT was also restricted to use triple-junction angles based on minimal polygons. On the other hand, the magnitude of the pressure error is more than 3 times any of the CellFIT errors. This is also likely due to the minimal polygon representation of cells, which lacks any information about cell edge curvature. If curvature information is retained by using non-minimal polygons for each cell – i.e., keeping all of the non-TJ nodes from the original forward model – then the system of equations is over-determined and both a Bayesian appraoch and VFM yield nearly identical high-quality solutions (tension and pressure errors of just 0.1% and 4.4%). Although this approach works for an inverse problem in which the original mesh is known, it requires extra care when applied to real images as there is an ambiguity in both how many non-TJ nodes to use and how to distribute them along the cell edges.

To test the response of the algorithms to noise, the angles and curvatures input to CellFIT were subjected to noise of various levels (Table 1 and Figs 1G and H). Appropriate noise levels were determined by having multiple trained users manually digitize portions of several images using basic digitizing tools. These tests showed that angles could be obtained by hand within approximately 5 degrees and radii of curvature within approximately 5% of their true values. We refer to this amount of uncertainty as noise level 5. Automated digitizing and segmentation tools may in time do better, so we include similar analyses for noise levels 1 and 2. To obtain the noise sensitivity values reported in Table 1, noise of the levels specified in the table was applied to the geometric data shown in Fig. 1B, as described in the table caption, and the tensions and pressures calculated by CellFIT were compared statistically to their corresponding ground truth values (Fig. 1D). All of the polyarc CellFIT methods performed similarly, with tension and pressure errors around 17–20% and 23–25% at noise level 5. Maintaining curvatures for the edges, but calculating the TJ angles using straight lines between triple junctions significantly degraded the performance of this hybrid CellFIT, even at the lowest noise levels. Nonetheless, even with added noise, all of the CellFIT methods outperformed Bayesian force inference based on minimal polygon representations. In the example investigated here, the Bayesian inference errors were already large without added noise, and only increased slightly with modest amounts of added noise (noise level 5 corresponds to an error in each vertex position of approximately 5% of the mean cell radius).

To test the sensitivity of CellFIT to pixilation and digitization effects, like those associated with the analysis of real image data, we subjected the synthetic cell mesh (Fig. 1B) to a rasterization and re-digitization process. This involved four major steps: (1) draw each cell-cell boundary as a spline curve onto an image of a given size; (2) generate a watershed image from the outline array, using a labeling process to automatically identify separate regions; (3) re-label the resulting image so the regions match those in the original mesh; and (4) pass this re-labeled watershed image through a contour-generating algorithm to create a new mesh [50]–[52]. In doing this, the original mesh lines became jagged and the exact location of mesh points was obscured, similar to the complications involved in the analysis of real image data. Even when CellFit was applied to a relatively coarse 675×657-pixel re-digitized image, it proved robust (Fig. 1I), yielding normalized RMS tension and pressure errors of 18% and 48%, respectively.

The robustness of CellFIT was further tested by giving it data from subregions as small as single cells or even single TJs from the original patch. The quality of any tensions and pressures that could legitimately be calculated were not decreased, though the Standard Solutions varied slightly as a result of small-sample statistical effects.

As a final test, CellFIT was applied to the 2D bubble raft shown in Fig. 8 of Stein and Gordon [38]. This image was chosen because it had a range of bubble sizes and shapes and the bubble edges could be digitized easily. The resulting tensions were within 5% of each other, consistent with the uniformity expected in static bubble rafts, and since they were not particularly interesting to examine, are not shown here.

## Tools for Assessing Solution Quality

The fact that CellFIT works well on synthetic data is a necessary condition for its validity as a useful inverse method, but it is not sufficient. We thus introduce here a number of tools that are useful for assessing the quality of a set of forces inferred from experimental images. These tools are standard practice in many types of statistical analysis, but not yet in force-from-shape analyses.

Condition numbers are the first of these tools, and the condition numbers for each of the geometric matrices **G** *_γ_* and **G** *_p_* is defined as the ratio of the largest to smallest singular values. If one were solving two equations in two unknowns, the condition number would provide an indication of whether the lines representing the equations were nearly normal to each other – thus yielding robust solutions, or nearly parallel to each other – yielding solutions that would be extremely sensitive to the exact positioning of the lines, noise and solver errors. Low condition numbers indicate a well-conditioned set of equations while high values indicate an ill-posed problem [53].

In force inference methods, both the tension and pressure equations have a rank deficiency of one arising from the unknown scaling of tensions and the unknown offset of the pressures (which would yield one singular value equal to zero and thus an infinite condition number). This rank deficiency is however accommodated by augmenting the geometric matrices with constraints on the mean tension and pressure (Equations 7 and 12). Nonetheless, even with such constraints, a number of situations can conspire to make the augmented matrices nearly singular and the inverse problem ill-conditioned. For example, cells with straight edges and certain regular or circle-inscribed geometries can have exactly compensating pressures and edge tensions [31], [37] or the entire cell patch can have nearly compensating radial gradients in the cell pressures and edge tensions. Such ill-conditioning is very common in force-inference methods that use polygonal cell approximations. Although problems related to specialized geometries, such as perfectly circular cells, could theoretically arise even when curved edges are allowed, we have not encountered them in practice. If such problems did arise, a high condition number would warn the user of ill conditioning. Inspection of the vector(s) corresponding to the zero or near-zero singular value(s) could then identify the problematic modes. In the experimental examples that follow, condition numbers for the Tension and Pressure Equations are indicated in the associated figure captions.

The second tool makes use of the residuals,
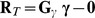
(17)and
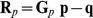
(18)of the tension and pressure equations, respectively. These residuals provide an indication of how well the calculated solutions satisfy the original equations. In general, the solutions are not exact because determination of edge curvatures and TJ angles from images introduces error. The Tension Equation residuals indicate the degree to which the *x*- and *y*-components of the least-squares or other “best” solution forces are out of equilibrium at individual TJs. They thus provide a measure of any inconsistencies in the calculated tensions. The residuals of the Pressure Equations provide similar information, but for pressures acting across cell-cell interfaces. In the experimental examples that follow, we visually report the residuals as thin lines radiating from each TJ (vectorial tension residuals) or subtending each cell-cell interface (scalar pressure residuals). The tension residuals are normalized by the average edge tension 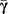 and scaled on the image so that a normalized residual of one has a length equal to the average cell radius. Pressure residuals are converted into forces by multiplying each by the chordal length of the edge on which it acts and then normalizing and scaling them against 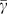 exactly like the tension residuals. If small regions of the cell sheet display exceptionally large tension or pressure residuals, these residuals could suggest digitizing errors or the presence of local forces such as traction forces that violate the assumption that shape is determined solely by edge tensions and intracellular pressures.

Finally, to assess the expected reliability of individual tensions or pressures, we report the conventional standard errors derived from the covariance matrix [46]. These standard errors are plotted below each tension/pressure map as error bars in sorted plots of the edge tensions and pressures. These plots convey the overall uncertainty in the tension and pressure estimates. For example, if one wanted to assess the reliability of a particular inferred force, such as the tension along an edge that forms part of a tissue boundary, its individual standard error value could be examined.

## Inferring Cellular Forces in Biological Systems

**Figure 5:**
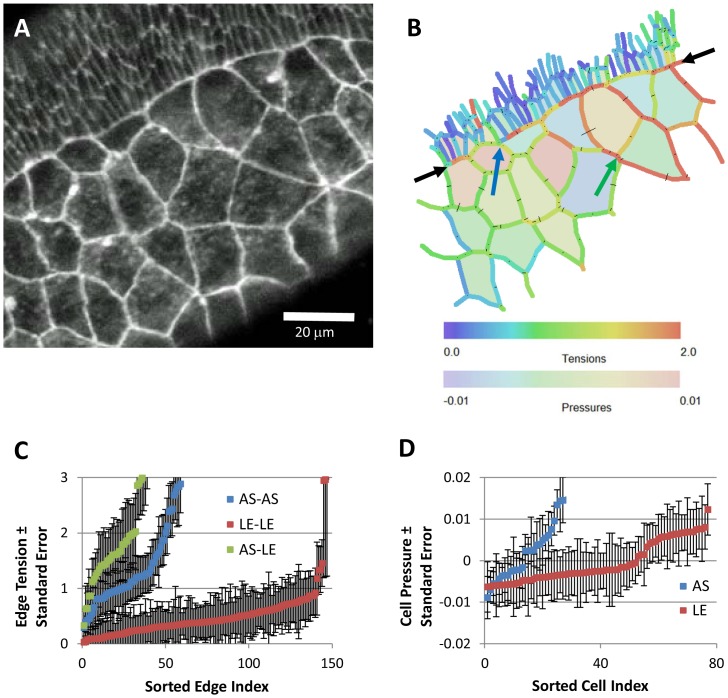
CellFIT analysis of cells near the amnioserosa/lateral epidermis boundary during early dorsal closure in a living *Drosophila* embryo, as imaged in (A) and with inferred Standard Tensions and Pressures illustrated in (B) according to the color bars. The amnioserosa is visible in a wide band from the lower left-hand corner towards the upper right, while the lateral epidermis, identifiable by its smaller cells, is confined to a large triangle in the upper left corner. The boundary between these two tissues is indicated by the black arrows. The blue and green arrows point to features discussed in the text. Overall, the CellFIT equations were very well conditioned – having tension and pressure condition numbers of 30.3 and 15.6, respectively. The tension and pressure residuals are shown in (B) as thin lines emanating from each triple junction and bisecting each cell edge, respectively. These residuals are scaled so that a residual equal to the mean tension has a length equal to the mean cell radius. Even at this scaling, the residuals are generally quite small and many are barely discernable. Finally, confidence limits are shown for individual tensions and pressures, (C) and (D) respectively, by boundary type. The points and bars indicate best estimate +/− one standard error (based on the covariance matrix), respectively. These confidence limits are a significant, but modest, fraction of the inferred tensions and pressures. Prior investigations have suggested the existence of a uniform high-tension purse-string along the edge of the amnioserosa, but CellFIT reveals a more complex and interesting scenario. See text for details.

Having validated the CellFIT algorithms using synthetic data and developed tools for assessing individual analyses, we can now use CellFIT to learn about the forces at work in several biological systems. To begin (Fig. 5), we consider the region where amnioserosa (AS) cells contact surrounding lateral epidermis (LE, the smaller cells in the upper left corner) during early dorsal closure in *Drosophila* embryos (Bownes stage 13). Conventional wisdom is that the cell edge tensions are modest everywhere except along the AS/LE boundary, where they are higher.

We collected confocal fluorescent images of these cells in living embryos that ubiquitously expressed E-Cadherin-GFP [54]. These cells have been investigated extensively and previous studies have found evidence for a strong pursestring force along this interface [55], [56]. If the tensions in individual cell edges were known, however, what additional information might a CellFIT analysis provide? Figure 5A shows an image of a representative sample of these cells and 5B shows the corresponding CellFIT Standard Solution for this image. As the figure caption shows, the Tension and Pressure Equations had low condition numbers (compared to the orders-of-magnitude higher values obtained from approaches based on straight edges), and the solution residuals are so small that they can be difficult to see in the figure. Both of these classes of information give reason for confidence in the calculations.

CellFIT reveals a more complex situation than is typically presented. For example, edge forces in individual cells change significantly around their perimeters and they vary considerably from one cell to the next. The intracellular pressures also show a range of values within each tissue. Although this should not be very surprising given that substantial variations occur in the detailed geometries of individual cells, a method like CellFIT is required to reliably identify, map and study this sort of mechanical variability. It cannot be obtained from the kinds of pointwise data that current experimental techniques provide. The edge tension plots in Figure 5C show that all three classes of cell-cell interfaces – LE/LE, AS/AS, and AS/LE – vary over the same wide range, but the median edge tensions differ, highest for AS/LE interfaces (1.6), lower for AS/AS interfaces (1.1), and lowest for LE/LE interfaces (0.4). This ordering is qualitatively consistent with previous laser ablation experiments, and within the low end of their quantitative estimates [57], [58].

A close examination of the AS/LE boundary shows that this low estimate is warranted because the interface is not consistently straight (at the cellular scale). Indeed, near the blue arrow, the AS/LE interface approaches the side of a cell in such a way that a large tension cannot exist along that interface at that point. The tension along the AS/LE boundary does, however, build up toward the right from that point as a consequence of what could be considered to be shear forces brought about in part by angled impinging edges. An examination of the standard errors of the edge tensions in this area shows that they are no higher than the average values, suggesting that the confidence level on these edges is high.

In addition, CellFIT shows a roughly 3-fold difference in AS/AS tensions when compared to LE/LE tensions. This difference is required if the outward-directed forces in the closely-spaced LE/LE edges of the ectoderm are to balance the inward forces produced by the more widely spaced and fewer in number AS/AS edges. As noted in previous estimates of the force ladders [54], these tensions are in approximate proportion to their spacing along the AS/LE boundary.

Even within the amnioserosa, the edge tensions are seen to vary considerably, and this result is consistent with these cells' local dynamic contractions. Examination of the angles and forces at particular TJs suggests forces that are indeed in balance and a revealed variability in edge tensions that is real. For example, at the closely spaced TJs indicated with a green arrow, four boundaries nearly meet at a quad junction with all angles near 90°. For equilibrium to occur in this special geometry, the tension along any one edge must be essentially the same as that in its extension on the other side of the cross. Other specialised geometries can exist, including ones that require certain edges to carry zero force, and a discussion of them can be found in structural analysis texts that discuss the method of joints [59].

**Figure 6:**
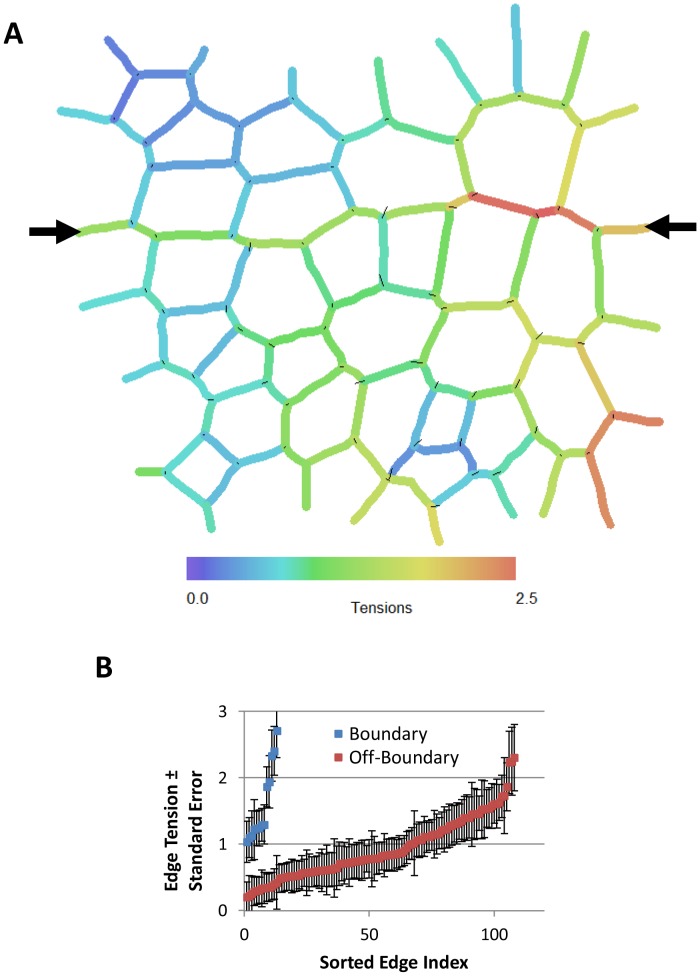
CellFIT analysis of a wing imaginal disc from a living *Drosophila* larva. (**A**) shows the inferred Standard Tensions based on Fig. 4D of Reference [4]. The tension equations are well conditioned (condition number of 11.1), the confidence limits are acceptable and the force residuals are a bit larger than for the previous case. The black arrows in (**A**) indicate the boundary between compartments within the imaginal disc. (**B**) shows that the forces along that boundary tend to be higher than those elsewhere (see also text). Pressures were not calculated as boundary curvatures could not be obtained from the source image.

As a second example, we consider imaginal discs in *Drosophila* larvae, which have also attracted much attention. Laser ablation experiments have shown that tension in the edges along the dorsal-ventral compartment boundary are approximately 2.5 times as strong as those in other nearby edges [4]. Figure 6 shows a CellFIT analysis of a wing imaginal disc tissue previously segmented using straight edges in Fig. 4D of the Dahmann paper [4]. Again, significant variability is found in the edge tensions. Intracellular pressures were not calculated as the cells were approximated by minimal polygons and so the edge curvatures needed to construct pressure equations were unknown. Although the magnitudes of the forces vary along the boundary, the ratio of the in-boundary to out-of-boundary tensions remains sensibly constant at approximately 2. Considering the errors associated with the use of straight edges as provided in their figure rather than curved edges (Table 1), this is consistent with their measured force ratio.

**Figure 7:**
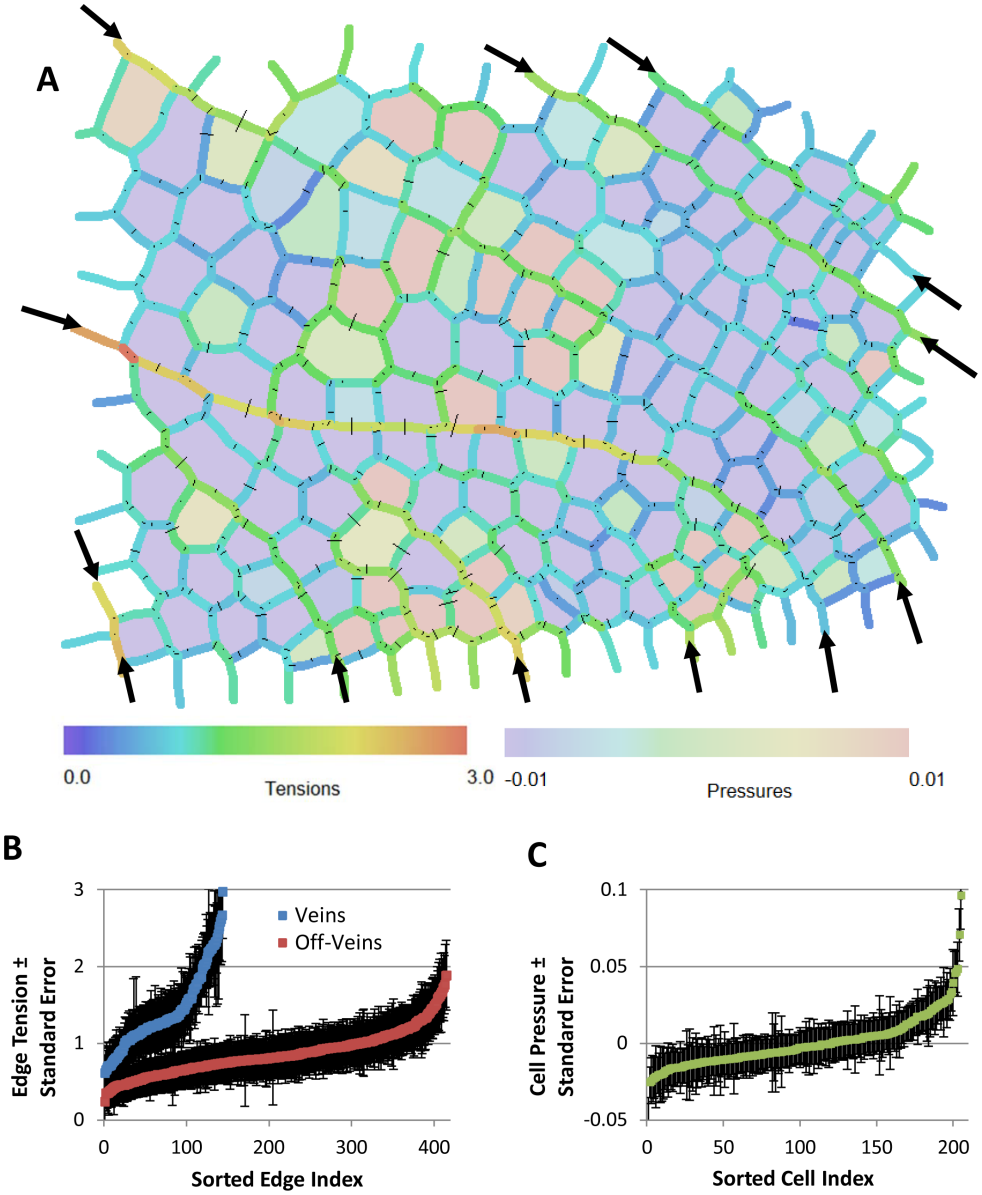
CellFIT analysis of an image of a dragonfly wing, published as Fig. 162 in Thompson's *On Growth and Form* [60]. (A) shows the inferred Standard Tensions and Pressures. The CellFIT equations were well conditioned (tension and pressure condition numbers of 16.7 and 13.9 respectively), the residuals are quite small, and the confidence limits for the tensions and pressures are acceptable (B and C). As noted in the text, the tensions along the veins (indicated with arrows) are significantly higher than those along the other cell edges. This example shows that CellFIT can extract useful information from historical images or from fixed or otherwise non-living tissues.

As a final example, we consider the historical dragonfly wing image reported by D'Arcy Thompson [60]. A CellFIT analysis (Fig. 7) reveals the tensions along the wing veins to be 1.6 times higher than those between other cells, and it shows noticeable tension variability along the veins and within off-vein groups of cells, findings consistent with the other two examples reported here. This example also suggests that if a particular specimen preparation method does not alter TJ angles and edge curvatures, CellFIT can used to infer the forces that were acting in the living state.

## Discussion

The last several decades have seen an increasing focus on cell and tissue mechanics and recognition of the important complementary role they play to genomic and protein analyses. Two of the most promising nascent technologies are force-reporting molecules [20] and force-from-image techniques like the one presented here. Both offer the important prospect of force maps obtained from single specimens, thus overcoming the challenges associated with the high natural variability found in biological specimens (often in the range of 30%). Indeed, only by constructing such maps can one identify and examine cell-to-cell tension variations like those reported here. The 2-fold to 20-fold variation found by CellFIT within a presumptively homogeneous group of cells may seem high, but individual cells in a group have been shown to have differences in gene expression and shape variability of this scale [61]. This degree of variability is also consistent with the tension variation of 30% found in tissues [11] and whose bulk properties can be considered to be a kind of statistical average of their individual cell properties.

Unlike some of the earlier force-from-image approaches [31], [32], CellFIT can be applied to single images, and forgetting factors and other techniques that involved multi-step data can be avoided. This advance removes the correspondence problems previously associated with mitosis, apoptosis and other events that change the topology of the cellular grid. Another important consequence is that the viscosity of the cytoplasm is no longer assumed to provide significant forces, and advanced finite element methods are no longer required to do the associated calculations. Also, because the tension and pressure equations are separated in the formulation presented here, it is possible to find tensions only. This feature is important when the technique might be applied to historical images where limited image resolution or other factors might make curvatures difficult or impossible to determine. Furthermore, if intracellular pressures are not required in a certain application, effort need not be expended on determining edge curvatures, *per se*, unless they are used to improve TJ angles.

The concept of Standard Solutions makes it clear how tensions and pressures in any tissue are related to each other and how the specific solution associated with a particular application can be determined uniquely as long as the scale factor α and pressure offset β can be found. One could envision the information needed to ascertain these values coming from sources such as aspiration experiments, AFM-like measurements, force-reporting molecules or one of the other techniques mentioned in the Introduction.

The mathematical tools presented here allow the quality of CellFIT solutions to be evaluated from a number of perspectives. For example, the conditioning of the Tension and Pressure Equations can be assessed by examining their respective condition numbers. The standard error of individual tensions and pressures can be ascertained using covariance matrices, and the degree to which individual TJ equilibrium and pressure differences have been satisfied can be tested by examining the residuals of their associated equations.

To ascertain the full range of situations in which the present equations are appropriate will require research beyond the scope of the present study. That research may involve finite element modeling of synthetic tissues subject to a wide range of different driving forces, and development of CellFIT equations with the ability to discriminate between a broader range of driving forces, such as tractions and stress fiber forces. The equations presented here may also be useful for analyzing other kinds of systems such as bubble rafts, spider webs and net-like cable systems, and they could be adapted to 3D applications.

As CellFIT is used with new or expanded conceptual models, it might be useful to append letters to denote the driving forces assumed [37]. The present analysis might be called CellFIT-TP since it assumes the action of tensions and pressures. Models that include viscosity might have a V appended while others might assume anisotropic area contractions (A) as from stress fibers, substrate tractions (S) or various far-field boundary conditions (B), and three-dimensional analyses might include the number 3. The more complicated situations encompassed by these approaches will likely demand that more terms be added to the General Solution and that more data from outside the images be used to correctly determine the contributions that each makes to the total forces present.

One could envision CellFIT being used alongside AFM, laser ablation or other force-measuring techniques, with each complementing and validating the other. It might also function in conjunction with studies of ultrastructure and gene expression, aiding our understanding of how these factors ultimately give rise to particular patterns of cell and tissue organization in embryos, normal and diseased tissues, and engineered organs.
